# Daily Monitoring of D-Dimer Allows Outcomes Prediction in COVID-19

**DOI:** 10.1055/a-1709-5441

**Published:** 2022-01-24

**Authors:** David M. Smadja, Olivier M. Bory, Jean-Luc Diehl, Alexis Mareau, Nicolas Gendron, Anne-Sophie Jannot, Richard Chocron

**Affiliations:** 1 Innovative Therapies in Hemostasis, University of Paris, Paris, France; 2 Hematology Department and Biosurgical Research Lab (Carpentier Foundation), AP-HP, Georges Pompidou European Hospital, Paris, France; 3 Emergency Department, AP-HP, Georges Pompidou European Hospital, Paris, France; 4 Intensive Care Unit and Biosurgical Research Lab (Carpentier Foundation), AP-HP, Georges Pompidou European Hospital, Paris, France; 5 Medical Informatics, Biostatistics and Public Health Department, Centre de Recherche des Cordeliers, AP-HP, University of Paris, Georges Pompidou European Hospital, European Georges Pompidou Hospital, Paris, France; 6 Emergency Department, University of Paris, PARCC, Georges Pompidou European Hospital, Paris, France

Dear Editor,

Coronavirus disease 2019 (COVID-19) is associated with a prothrombotic phenotype, and D-dimer level at admission is a prognostic factor. 1 2 3 4 5 6 Some meta-analyses have tried to predict the outcomes of patients with COVID-19, including D-dimer levels, primarily those at hospital admission. 7 Most studies published to date have used baseline measurements or included participants with incomplete follow-up data. Few studies have been published about the dynamics of early changes in D-dimer levels in hospitalized patients with COVID-19 and their potential suitability for outcome assessment, that is, in-hospital mortality or disease worsening with intensive care unit (ICU) transfer. 8 9 However, D-dimer cutoff during follow-up to predict the outcomes and their involvement in daily clinical management of patients with COVID-19 is yet to be determined. 10

**Fig. 1:**
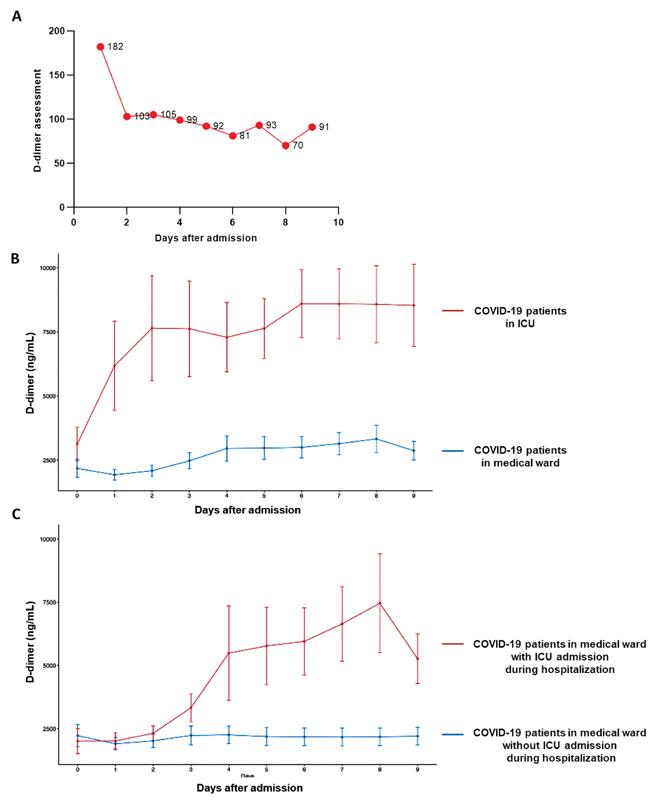
Daily monitoring of D-dimer levels and outcome prediction in coronavirus disease 2019 (COVID-19). ( **A** ) Number of D-dimer level assessments. ( **B** ) Temporal trend of D-dimer levels of critical and noncritical patients with COVID-19: Red line: patients admitted to the intensive care unit (ICU) directly after emergency department; blue line: patients admitted to a medical ward directly after emergency department. ( **C** ) Temporal trend of D-dimer levels of patients admitted to a medical ward after emergency department with and without ICU transfer during hospitalization. Red line: patients admitted to the ICU after hospitalization in a medical ward; blue line: patients who were hospitalized only in a medical ward.

In the retrospective study presented here, we monitored the daily D-dimer levels in a large cohort of 320 adult COVID-19-positive patients hospitalized at the Georges Pompidou European Hospital between February 1 and June 30, 2020 who underwent at least two D-dimer assessments during follow-up. We quantified D-dimer levels (Vidas D-dimers assay, Biomérieux, Marcy-Etoile, France; limit of quantification <45 ng/mL) during the first 9 days of hospitalization. The number of D-dimer assessments is shown in Fig. 1A . In our cohort, 213 (66.6%) patients were male, and 35 (10.9%) were obese; the median age was 66.5 years (interquartile range [IQR]: 56.8–77.0). This cohort included 212 (66.2%) patients with COVID-19 first hospitalized (after emergency unit) in a medical ward and 108 (33.8%) patients first hospitalized in the ICU. D-dimer levels were assessed four times (IQR: 2–7) per patient during the first 9 days of hospitalization. We decided to stop evaluation at day 9 for multiple reasons: first, during follow-up, the monitoring of D-dimer levels was not regular because it was at the discretion of the treating physician, and after day 9, the frequency of D-dimer measurements decreased significantly. Second, the two main outcomes (ICU referral and in-hospital mortality) occurred more frequently in the first 10 days of hospitalization. Because our study aimed to assess the predictive value of D-dimer monitoring for COVID-19 worsening and in-hospital mortality, we focused on the period before the occurrence of the event (ICU referral or in-hospital mortality). Finally, our observation period of 9 days still covered the median time to death observed in previous studies on the first wave of patients with COVID-19; for example, Valerio et al reported that the time to death was 7 days (IQR: 4–12 days). 8 Missing data were handled by imputation using a linear interpolation from observed values (approximation function of the *stats* package of R software).

In general, D-dimer levels during follow-up were higher in patients in the ICU than in patients in a medical ward ( Fig. 1B ). Of the 212 patients with COVID-19 directly admitted to a medical ward, 21.7% ( *n* = 46) had an ICU transfer during hospitalization, whereas 78.3% ( *n* = 166) stayed in the medical ward ( Table 1 ). Median time for transfer to the ICU from a medical ward was 9.0 days (IQR: 4.0–15.8). For these two populations, D-dimer levels were not significantly different during the first 3 days of hospitalization ( Fig. 1C ). Then, after day 4, we observed a significant increase in D-dimer levels only for patients transferred to the ICU ( *p* < 0.001 at day 4 using repeated measure analysis of variance with Bonferroni's correction; this difference remained significant from day 5 to 9), whereas for patients who stayed in the medical ward, daily D-dimer levels were not significantly different over time. To assess the ability of D-dimer monitoring in the first 9 days of hospitalization to predict outcomes (ICU referral or in-hospital mortality), we analyzed the ratio of D-dimer (RoD) levels defined as either the D-dimer value on the day of outcome occurrence or the highest value during the first 9 days (if the outcome did not occur) divided by the D-dimer level at admission. The RoD is the percentage change from baseline level; the percentage change is a simple concept that represents the degree of change over time. Thus, the RoD takes into account the difference between patients at baseline. Each patient has a different baseline D-dimer level, which varies widely according to COVID-19 severity.

**Table 1:** Clinical characteristics and ability of D-dimer monitoring in the first 9 days of hospitalization to predict outcomes (ICU referral or in-hospital mortality)

	Overall	Medical Ward			ICU
		Whole population	Patients who stayed in medical ward	Patients in medical ward; secondary transfer to ICU	Patients directly admitted to ICU
	*n* = 320	*n* = 212	*n* = 166	*n* = 46	*n* = 108
Age, years—median [IQR]	66.5 [56.8–77.0]	69.0 [57.0–79.3]	70.5 [57.0–80.0]	68.5 [56.3–75.0]	64.0 [55.8–70.0]
Female, *n* (%)	107 (33.4)	83 (39.2)	70 (42.2)	13 (28.3)	24 (22.2)
Male, *n* (%)	213 (66.6)	129 (60.8)	96 (57.8)	33 (71.7)	84 (77.8)
BMI ≥30 kg/m ^2^ , *n* (%)	35 (10.9)	20 (9.4)	11 (6.6)	9 (19.6)	15 (13.9)
Length of stay, days—median [IQR]	11.0 [5.0–23.0]	8.5 [3.0–16.0]	6.0 [1.3–12.0]	23.0 [14.0–34.5]	19.0 [10.0–35.0]
Length of stay in ICU, days—median [IQR]	15.0 [6.0–25.3]	10.0 [5.3–23.0]	–	10.0 [5.3–23.0]	16.0 [6.0–26.0]
Time from admission to ICU admission, days—median [IQR]	–	9.0 [4.0–15.8]	–	9.0 [4.0–15.8]	–
non-survivor *n* (%)	68 (21.2)	31 (14.6)	16 (9.6)	15 (32.6)	37 (34.3)
Time from admission to in-hospital death, days—median [IQR]	13.5 [6.8–20.0]	13.0 [7.0–20.0]	12.0 [5.5–15.3]	14.0 [10.5–25.5]	15.0 [6.0–20.0]
ICU referral prediction	RoD	+28%			–
	Adjusted HR * [95% CI], *p* -value	3.99 [3.02–5.25], <0.001			
In-hospital mortality prediction	RoD	+69%			+74%
	Adjusted HR * [95% CI], *p* -value	2.85 [2.49–3.26], <0.001			5.62 [4.15–7.60], <0.001

Abbreviations: BMI, body mass index; CI, confidence interval; HR, hazard ratio; ICU, intensive care unit; IQR, interquartile range; RoD, ratio of D-dimer.

Increase in RoD was evaluated using receiver operating characteristic curve analysis. If the outcome occurred during the first 9 days, RoD was defined as the ratio of D-dimer level on the day of outcome occurrence/D-dimer level at admission; if the outcome did not occur during the first 9 days, RoD was defined as the ratio of the highest D-dimer level during the first 9 days/D-dimer level at admission.

* Hazard ratio from Cox proportional hazard model adjusted for age, gender, BMI (< or > 30 kg/m ^2^ ).

Using Youden's index method, we identified different optimal thresholds for RoD: for patients with COVID-19 directly admitted to the ICU, a threshold of 74% increase in RoD was a predictor of in-hospital mortality (with corresponding area under the curve [AUC]: 67.5; 95% confidence interval [CI]: 57.9–70.4); for patients admitted in a medical ward, a threshold of 28% increase in RoD was a predictor of ICU referral (AUC: 77.0, 95% CI: 74.6–79.4) and a threshold of 69% was predictor of in-hospital mortality (AUC: 68.8, 95% CI: 65.4–72.2). Using Cox proportional hazard model adjusted for age, sex, and obesity, a significant association was found between the 74% RoD threshold and in-hospital mortality in patients with COVID-19 directly admitted to the ICU (adjusted hazard ratio [HR]: 5.62; 95% CI: 4.15–7.60, Table 1 ). For patients admitted to a medical ward, a significant association was found between the 28% RoD threshold and ICU referral (adjusted HR = 3.99; 95% CI: 3.02–5.25) and between 69% RoD threshold and in hospital-mortality (adjusted HR = 2.85; 95% CI: 2.49–3.26). To the best of our knowledge, this is the first description of D-dimer daily monitoring according to COVID-19 severity in a large cohort with the establishment of a useful cutoff for follow-up. Our findings suggest that a ∼30% increase in D-dimer levels in daily clinical evaluation predicts ICU referral and that a 70% increase predicts in-hospital mortality during medical ward stay. Thus, RoD may help physicians to monitor a patient more frequently or transfer them to another ward/unit with higher level of care.

Increased D-dimer level is a hallmark of COVID-19 severity, likely reflecting microthrombosis. Indeed, endotheliopathy associated with severe acute respiratory syndrome coronavirus 2 infection may explain coagulopathy, lung obstruction, and right ventricle overload. 2 3 11 Thus, early D-dimer monitoring may support the choice of the most appropriate anticoagulation regimen. Difference in D-dimer levels at admission is an important indicator 4 ; however, the course of change in D-dimer levels is relevant and may better predict outcomes, as demonstrated in the present study. Our study has several limitations: first, the identification of the optimal threshold value based on Youden's index has several limitations. We were unable to test the optimal threshold on a different cohort than the one in which it was derived; however, we compared different optimal cutoff points using several metrics and selected one that was most clinically relevant. Our ultimate goal was to maximize clinically meaningful D-dimer diagnostic performances to obtain a prognostic score. Our optimal threshold values need to be confirmed in an external cohort of patients with different clinical characteristics. Another limitation of our study is the absence of anticoagulation regimen adaptation or interaction. The study presented here was done during the first wave of COVID-19 pandemic while prophylactic dosing of heparin was used in all patients. Therapeutic and/or intermediate-dose prophylactic anticoagulation in patients with COVID-19 was tested and used after this period. We previously demonstrated that anticoagulation therapy before hospitalization was associated with a better prognosis. 12 Several randomized studies confirm this hypothesis of “earlier is better” for anticoagulation in COVID-19 course, 13 14 probably because early initiation of anticoagulation prevents onset of extensive microthrombotic processes. Daily monitoring of D-dimer levels to assess COVID-19-associated coagulopathy, mainly in the first few days of the disease, should be tested in dedicated clinical trials according to the initial anticoagulation regimen and its relevance to adjust the anticoagulant dose.

All in all, our findings indicate that higher D-dimer levels and modified kinetics are associated with ICU referral and in-hospital mortality in COVID-19. Thus, daily monitoring of D-dimer levels during hospitalization and their comparison with the D-dimer levels at admission are valuable in monitoring disease progression. Their predictive value should be verified in large studies testing the association between routine measurement of D-dimer levels and markers of endotheliopathy and inflammation.
